# Beyond the burden of cardiovascular and cancer mortality among adults: mental health as a cause of death

**DOI:** 10.1590/1516-3180.2019.1374.220819

**Published:** 2019-10-31

**Authors:** Paulo Andrade Lotufo

**Affiliations:** I MD, DrPH. Full Professor, Department of Internal Medicine, Faculdade de Medicina da Universidade de São Paulo (FMUSP), São Paulo (SP), Brazil.

In a previous editorial in this Journal this year, I addressed the temporal trends of the risk of death due to the most critical causes among Brazilians aged 45 to 64 years: circulatory disorders and neoplasms.^1^ The aim was to make comparisons with the patterns of heart disease and cancer deaths in the United States.^2^ The results were that cardiovascular diseases showed reductions in age-adjusted rates from 2000 to 2010 for men (-1.8% per year) and women (-2.2% per year). Over the same period, the death rates due to cancer stagnated for men and climbed for women (0.6% per year), mainly due to smoking-related neoplasm.^1^

One point of interest in this regard is to verify the pattern of mortality beyond cardiovascular diseases and cancer from 2000 to 2017 for the age stratum of 45-64 years. Four years ago, one report astonished public health managers and epidemiologists through showing that among white men aged 45-54 years, there were increases in the risk of death due to drug and alcohol poisoning, cirrhosis and suicide.^3^

To test the trends of death due to non-cancer and non-cardiovascular disease causes in Brazil, I firstly used the Tenth Revision of the International Classification of Diseases (ICD-10) to calculate which causes of deaths according to sex showed absolute increases in the numbers of events. From this, I identified in the Brazilian DATASUS system (http://datasus.saude.gov.br), 10 underlying causes of death with absolute increases over this period: viral hepatitis (ICD-10: B15-B19); HIV-related disorders (ICD-10: B20-B24); diabetes (ICD-10: E10-E14); mental disorders due to psychoactive drugs (ICD-10: F10-F19); chronic obstructive pulmonary disorder (ICD-10: J40-J47); cirrhosis due to alcoholic beverages (ICD-10: K70); renal disorders (ICD-10: N17-N19); road accidents (ICD-10: V01-V99); homicide (ICD-10: X85-Y09); and suicide (ICD-10: X60-X84). Secondly, I applied Poisson regression to test whether the death rates trends of these ten causes reached statistical significance.

For one group of causes (viral hepatitis, diabetes and chronic obstructive pulmonary disorder), despite the rise in the absolute number of cases, there were significant reductions per year in the mortality rates. For two other causes (road accidents and homicides), the risk of death did not change materially during this period.

On the other hand, five conditions showed significantly increased risks of mortality: HIV-related disorders, mental disorders due to psychoactive drugs (women), cirrhosis due to chronic alcoholic beverage intake (men), renal disorders and suicide. Table 1 shows that from 2000 to 2017, these five causes together accounted for an increasing proportion of all-cause deaths, from 6.7% to 10% (men) and from 3.4% to 5.9% (women). The pace of the increase in death rates for men was high for HIV-related disorders, use of psychoactive drugs, suicide and renal diseases.

**Table 1.** Proportional mortality (PM), annual percentage change (APC) and 95% confidence interval (95% CI) for five causes of death in Brazil with increasing trends, among people aged 45-64 years over the period from 2000 to 2017

Cause of death	Men		Women	
	PM 2000-17	APC (95% CI)	PM 2000-17	APC (95% CI)
HIV	1.1% to 1.6%	1.3 (0.9 to 1.8)	0.7% to 1.3%	2.3 (1.6 to 3.0)
Drug abuse	1.5% to 2.1%	1.0 (-0.0 to 1.9)	0.3% to 0.7%	3.1 (1.8 to 4.4)
Cirrhosis	1.9% to 2.8%	0.9 (0.4 to 1.4)	0.4% to 0.6%	0.8 (-0.1 to 1.8)
Renal disorders	1.3% to 1.9%	1.4 (0.9 to 1.9)	1.6% to 2.6%	1.9 (1.3 to 2.5)
Suicide	1.0% to 1.5%	0.7 (0.2 to 1.2)	0.4% to 0.7%	2.0 (1.5 to 2.6)

Note: the numbers of deaths and populations were obtained from the Brazilian public health system DATASUS, 2019, and the annual percentage change was obtained by applying Poisson regression, available through the Joinpoint regression software 4.7.0.0 (https://surveillance.cancer.gov/joinpoint/; downloaded in February 2019).

The increase in deaths caused by renal diseases can be better understood through the increase in the incidence of end-stage kidney failure.^4^ Deaths relating to HIV can be explained partially as due to prolongation of the lifespan of the generation with high incidence of the infection who began to receive highly active antiretroviral therapy HAART (in the early 1990s), among whom there are now higher mortality rates among older men; and partially because of HIV cases among intravenous drug users.^5^

The combination of greater risk of death due to use of psychoactive drugs, suicide, cirrhosis due to alcohol intake and a proportion of human immunodeficiency virus (HIV) cases relating to intravenous drug indicates the existence of a cluster of mental health problems. This situation in Brazil is therefore relatively similar to what has been described in the United States as deaths due to overdoses of illicit drugs, cirrhosis and suicide.^6^

If these trends are real, as has been proven in the United States, and given that I do not have any means to refute that this is also valid in Brazil, then epidemiologists, anthropologists and social scientists face a big challenge in trying to decipher what is going on in our society.
